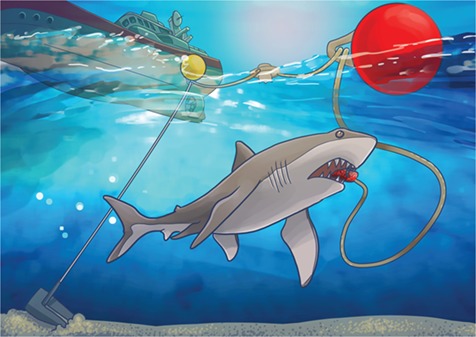# ‘SMART’ shark capture technology may keep beachgoers and sharks safe

**DOI:** 10.1093/conphys/coz087

**Published:** 2019-12-04

**Authors:** Christine L Madliger

**Affiliations:** Carleton University, 1125 Colonel By Dr., Ottawa, Ontario, Canada, K1S 5B6

The relationship between humans and sharks can be complex. However, fatal interactions with people are rare, accounting for less than six deaths worldwide per year. Yet, such events are often highly publicized and can negatively shape the public’s perception of sharks. Finding ways to eliminate negative interactions is, therefore, beneficial to both sharks and humans.

Traditional fishing methods, like nets and large baited hooks called drumlines, are often lethal to the sharks they catch, including both targeted species and other non-target endangered species. But a new type of ‘SMART’ (Shark Management Alert in Real Time) drumline has the potential to keep unwanted sharks far from beaches without causing the animals harm. While it is not yet clear how effective these new drumlines will be at minimizing shark interactions with humans, a group of researchers in Australia have begun testing the effects of this technology on shark physiology.

The SMART drumline consists of a hook and bait, just like a traditional drumline. The exciting addition is a GPS-enabled buoy that is activated the moment a shark takes the bait. A signal is transmitted to scientists or fishers so they can quickly respond to the capture location, remove the animal from the hook and release the shark.

In a recent study, Rick Tate and his colleagues aimed to determine the amount of time that white sharks could remain captured on SMART drumlines without facing negative consequences. In the area of Australia where this work was completed, white sharks are protected but thought to be the source of one third of all shark bites since 2000, making them an ideal study species. Tate’s team assessed 47 captured white sharks by analyzing a blood sample for a variety of metabolites known to change when animals are stressed or physically exhausted. Specifically, they investigated how these blood markers changed in relation to the length of time a shark had been waiting on the line to be released.

The researchers found that only two metabolites, lactate and aspartate aminotransferase, increased with the length of time a shark was left on a line. Both metabolites have been shown to rise when animals have to expend a lot of energy, which may happen if a shark is attempting to escape from the hook after consuming the bait. However, the 16 other metabolites they measured, including many related to stress, did not change over 75 min of capture time. This result could indicate that SMART drumlines may be a relatively low-stress capture method if short response times are used. However, future research is needed because longer response times may be more realistic but could also result in greater or more long-term stress for captured animals.

It is also important to determine how other species of sharks respond to being captured and to track released sharks to monitor survival, as delayed mortality is an issue with some species following exhaustive exercise. However, findings from this study will at least give managers a starting point—a conservative response time in which to locate and release sharks from drumlines—to ensure that both sharks and beachgoers remain safe.

Illustration by Erin Walsh; Email: ewalsh.sci@gmail.com

**Figure:**